# Sensory feedback-dependent coding of arm position in local field potentials of the posterior parietal cortex

**DOI:** 10.1038/s41598-021-88278-5

**Published:** 2021-04-27

**Authors:** Paul VanGilder, Ying Shi, Gregory Apker, Christopher A. Buneo

**Affiliations:** School of Biological and Health Systems Engineering, Arizona State University, P.O. Box 879709, Tempe, AZ 85287-9709 USA

**Keywords:** Sensorimotor processing, Biomedical engineering, Motor control

## Abstract

Although multisensory integration is crucial for sensorimotor function, it is unclear how visual and proprioceptive sensory cues are combined in the brain during motor behaviors. Here we characterized the effects of multisensory interactions on local field potential (LFP) activity obtained from the superior parietal lobule (SPL) as non-human primates performed a reaching task with either unimodal (proprioceptive) or bimodal (visual-proprioceptive) sensory feedback. Based on previous analyses of spiking activity, we hypothesized that evoked LFP responses would be tuned to arm location but would be suppressed on bimodal trials, relative to unimodal trials. We also expected to see a substantial number of recording sites with enhanced beta band spectral power for only one set of feedback conditions (e.g. unimodal or bimodal), as was previously observed for spiking activity. We found that evoked activity and beta band power were tuned to arm location at many individual sites, though this tuning often differed between unimodal and bimodal trials. Across the population, both evoked and beta activity were consistent with feedback-dependent tuning to arm location, while beta band activity also showed evidence of response suppression on bimodal trials. The results suggest that multisensory interactions can alter the tuning and gain of arm position-related LFP activity in the SPL.

## Introduction

Multisensory (or multimodal) integration (MSI) is crucial for sensorimotor function, particularly when estimating the state of the body (i.e. the position and velocity of relevant body segments) and planning movements. Combining multiple sensory cues provides a means of overcoming inherent noise in the sensory systems and reduces uncertainty in state estimates^1^. This has been demonstrated across a variety of behavioral domains including target localization, object recognition, navigation, and limb movement^2–7^. For example, during reaching movements, visual and proprioceptive cues are thought to be combined with state estimates derived from efference copies of motor commands and a forward model to form more precise localization of the limb^8,9^. However, it is still unclear how sensory cues are combined in the brain. Although early neurophysiological studies of subcortical neurons emphasized responses to weak unimodal stimuli^10^, which tends to result in multisensory enhancement of spiking activity, more recent investigations of the cerebral cortex suggest subadditivity (including suppression) may be more common^4,11–14^. Importantly, such multisensory suppression has been shown to be associated with greater information transmission^11^ and improved decoding accuracy^14^.

More recent work indicates that MSI may manifest in the brain through other mechanisms as well. Changes in neuronal spike timing or variability have been observed during multisensory interactions^11,14–17^ and analyses of local field potentials (LFPs) in both the time and frequency domains have provided evidence for both multisensory enhancement and suppression^18–23^. Regarding visual-proprioceptive interactions specifically, effects of multisensory interactions on spiking activity in the superior parietal lobule (SPL) appear to be dependent upon behavioral context, recording site, and neural coding scheme (rates vs spike timing)^14,17,24^. However, little information exists regarding the effects of such interactions on LFPs in the SPL or elsewhere.

We have previously characterized multisensory interactions in a population of SPL neurons as non-human primates performed an arm position maintenance task with unimodal (proprioceptive) or bimodal (visual-proprioceptive) sensory feedback^14,17^. This task allowed us to study visual-proprioceptive interactions under quasi-static rather than highly dynamic conditions, which pose greater interpretational challenges. Moreover, study of the neural correlates of arm position maintenance have been relatively ignored, despite evidence that they may only partially overlap with those involved in movement^25,26^. Regarding effects of multisensory interactions, we found that, relative to unimodal conditions, neuronal firing rates were largely suppressed under bimodal conditions. In addition, some neurons exhibited beta (13–30 Hz) oscillatory spiking under only one set of sensory conditions (unimodal or bimodal), while others did so under both conditions. In the current study, we examined LFPs recorded during these same experiments. We hypothesized that patterns of enhancement and suppression observed in spiking activity would be reflected in evoked LFP responses, i.e. these responses would be suppressed on bimodal trials relative to unimodal trials. In addition, we expected to see modulations of LFP power in the beta band that mirrored those observed in the spike spectra, i.e. beta power would be enhanced at individual recording sites under one or both sets of conditions.

## Methods

### Experimental subjects and paradigm

All experimental and veterinary care procedures were approved by the Arizona State University Institutional Animal Care and Use Committee and conducted according to the U.S. Public Health Service Policy on Humane Care and Use of Laboratory Animals (Public Law 99–158) and the Guide for the Care and Use of Laboratory Animals (National Academy Press, 1996). Environmental social enrichment, housing, and feeding procedures also conformed to institutional standards, which are AAALAC International accredited.

We have described the experimental paradigm and apparatus in previous reports but provide an overview here^14,17^. Briefly, two rhesus macaques (‘X’ and ‘B’) were trained to make reaching movements within a semi-immersive 3D virtual reality environment displayed on a 3D monitor and projected onto a mirror in their fields of vision. The monkeys made center-out reaches to eight peripheral targets and maintained their hand location at these targets with or without visual feedback. The mirror blocked the view of each monkey’s actual arm, but visual feedback of hand location was provided as a spherical cursor within the virtual environment. An active motion tracking system (Phoenix Technologies Inc.) monitored arm movements via LED markers placed on each monkey’s wrist. Eye movements were tracked using a remote optical tracking system (Applied Science Laboratories, Inc.). At the start of each trial, an animal had to align the arm cursor with the starting location, which appeared as a green sphere presented in the center of the virtual workspace. Once this location had been maintained for 500 ms (baseline period), one of the peripheral reach targets was pseudorandomly presented, serving as the “go” cue to begin the reach (movement period). When the peripheral target was acquired, an animal performed a saccade back to the starting location. This began the “static holding period,” where the animal maintained its hand location at the peripheral target while fixating at the starting location for 800–1200 ms. During the static holding period, visual feedback of the arm cursor was allowed on half the trials (bimodal condition) and removed on the remainder (unimodal condition). Spherical behavioral windows with radii ranging from 2 to 2.4 cm surrounded the reach targets and a behavioral window with a radius of ~ 6.5° of visual angle surrounded the fixation point. Trials were deemed successful if the animals acquired both the reach targets and fixation point and maintained position within these windows for the remainder of the trial. Animals completed five successful trials to each target in both sensory conditions and target locations were pseudorandomly varied on a trial-by-trial basis.

### Data acquisition

We analyzed evoked LFP responses from 170 recording sites (97 from monkey X, and 73 from monkey B) located within the superficial cortex of the superior parietal lobule (area 5). Note that this is less than the number of recording sites reported in Shi et al.^14^ and VanGilder et al.^17^ due to technical issues that corrupted the signals at some sites. LFPs were recorded acutely using varnish-coated tungsten microelectrodes (~ 1–2 MΩ at 1 kHz). LFPs were separated from the spike data after amplification by low-pass filtering at 300 Hz and were sampled at 1 kHz before saving to disk with the associated behavioral data (Multichannel Acquisition Processor, Plexon Inc.).

### Data analysis

All analyses were conducted in MATLAB (MathWorks, Natick, MA). For all statistical analyses, an alpha of 0.05 was used.

#### Evoked potentials

LFP data underwent two stages of preprocessing. The Chronux toolbox (Bokil et al.^27^, http://chronux.org) was used to remove line noise and slow voltage fluctuations caused by electrical transients that may cause a slow “drift” of the signal. Line noise was removed using Thomson’s regression method to detect and remove 60 Hz sinusoids and any harmonics from the data^27,28^. A sliding-window linear regression procedure was used to remove the slow voltage drift wherein a least-squares trend line was fit to the signal within each successive temporal window. Subsequently, the best fitting trend lines in each window were then weighted and combined to estimate the slow fluctuation, which was then removed from the data signal.

To examine the effects of hand location and sensory condition on evoked responses, the filtered LFP signals were first aligned to the start of the holding period. For each hand location, the mean LFP response for each trial and sensory condition was squared, and then averaged over the holding period time window. The square root of this quantity (RMS) was then compared across final hand locations and sensory conditions^29,30^. Specifically, a two-way ANOVA (factors: 8 hand locations, 2 sensory conditions) was used to assess the effects of sensory condition and reach direction on the mean evoked LFP response at individual recording sites during the baseline, movement, and holding periods.

Using the same framework from previous experiments^14,31^ an enhancement/suppression index was also computed for the evoked LFP responses obtained at each site. First, a preferred hand location was determined. Following convention used in previous studies, the preferred location was defined as the hand location with the largest trial-averaged evoked response in the Unimodal condition^14,29^. Next, LFP responses were averaged across trials for the preferred hand location for both Bimodal and Unimodal conditions. Enhancement/suppression indexes were computed as follows:

1 $${\text{INDX1}} = \frac{{B_{peak\_U} - U_{peak\_U} }}{{U_{peak\_U} }} \times 100$$

where B and U refer to the trial-averaged evoked responses for the preferred hand locations (as defined by the Unimodal condition). For this index, positive values indicate enhancement and negative values indicate suppression of LFP responses under bimodal conditions. Suppression or enhancement was considered to be statistically significant based on the result of the ANOVA performed on the RMS values. We also calculated a second index (INDX2) to account for the possibility that some enhancement/suppression might arise from differences in tuning between the two conditions, where the bimodal peak was calculated using the preferred hand location as defined by the bimodal condition:

2 $${\text{INDX2}} = \frac{{B_{peak\_B} - U_{peak\_U} }}{{U_{peak\_U} }} \times 100.$$

We also analyzed population-level differences in the evoked responses. For this, evoked responses were first normalized by baseline activity then averaged across trials and recording sites for the preferred location in both conditions. For these analyses, a nonpreferred location was also determined and was defined as the hand location with the lowest trial-averaged evoked response in the Unimodal condition. T-tests were used to determine if the evoked responses differed between Bimodal and Unimodal conditions, and between preferred and nonpreferred locations for each condition.

#### Spectral analysis

Temporal structure in the LFPs was analyzed using the multitaper spectral estimation technique. For each trial, we used 9 data tapers and a time-bandwidth parameter of 5, providing a spectral resolution of 6.25 Hz. For each recording site, trial-averaged power spectra with jackknife error bars were computed for each reach direction/hand location and condition. At the population level, spectra for each recording site were normalized by the average baseline power (− 500–0 ms prior to target onset) before averaging across sites. Population spectra error bars were derived from the jackknife standard error across recording sites. All analyses were performed using custom MATLAB programs supplemented by the Chronux toolbox^27^ for multitaper spectral analyses.

We also analyzed the average LFP spectral power within each of the following frequency bands: delta (0–4 Hz), theta (4–8 Hz), alpha (8–12 Hz), beta (13–30 Hz), and gamma (30–90 Hz). For our previous analyses of spike times, we focused on the beta band due to the prevalence of strong oscillatory activity in this range in posterior parietal areas during various tasks^32–34^ and its reported role in linking large-scale cortical networks during the maintenance of sensorimotor state^35,36^. As a result, we focused most of our attention on the beta band in the present investigation as well. As with the evoked responses, for each recording site a two-way ANOVA (hand location, sensory condition) was conducted to assess the effects of reach direction/hand location and sensory condition on the power in a given band. At the population level, T-tests were used to test for differences between population spectra associated with each sensory condition, as well as between preferred/nonpreferred locations for each condition.

## Results

### Behavior

Analyses of behavioral data were previously reported^14^ but will be summarized here. The experimental paradigm maximized the likelihood that final arm locations would be the same during both Bimodal and Unimodal conditions of the task. This was to ensure that any observed changes in neural responses could be interpreted as resulting from interactions between sensory cues. Mean endpoint locations and variances along the horizontal, vertical and depth axes did not differ significantly between sensory conditions, suggesting that during the static holding period, hand location was largely identical. The results of the current study were interpreted within this context.

### Time domain

**Figure 1:**
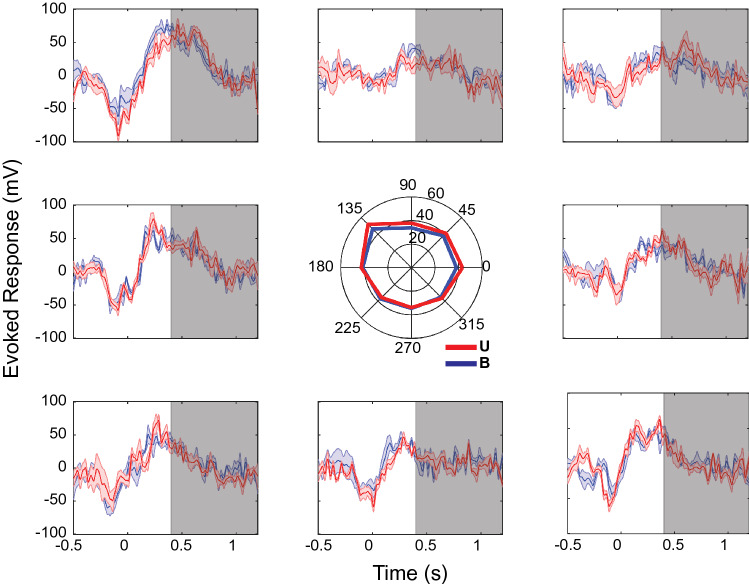
Evoked LFP responses at a recording site with only significant effects of hand location during the holding period. Each panel corresponds to one of the 8 reach targets. Responses were averaged across trials (N = 5, with jackknife error bars) aligned to reach target acquire. Grey box corresponds to the static holding period (0.4–1.2 s) after target acquire.

We performed a two-way ANOVA (factors: hand location, sensory condition) on the mean evoked LFP responses recorded at individual sites during the baseline, movement, and holding periods (see Table 1 in Supplementary Materials). In general, main effects of hand location were common during the 800 ms static holding period, though at a smaller number of sites (43/170; ~ 25%) than during the movement period (57/170; ~ 34%). Figure 1 shows the LFP evoked responses at an exemplary recording site that demonstrated significant effects of hand location during the holding period. The voltage traces for both sensory conditions were largely similar at each hand location, though there were clear differences among the responses at the eight target locations. Looking at the central polar plot, mean LFP evoked potentials were greater for hand locations up and to the left of the starting location, with a maximal response at 135°.

**Figure 2:**
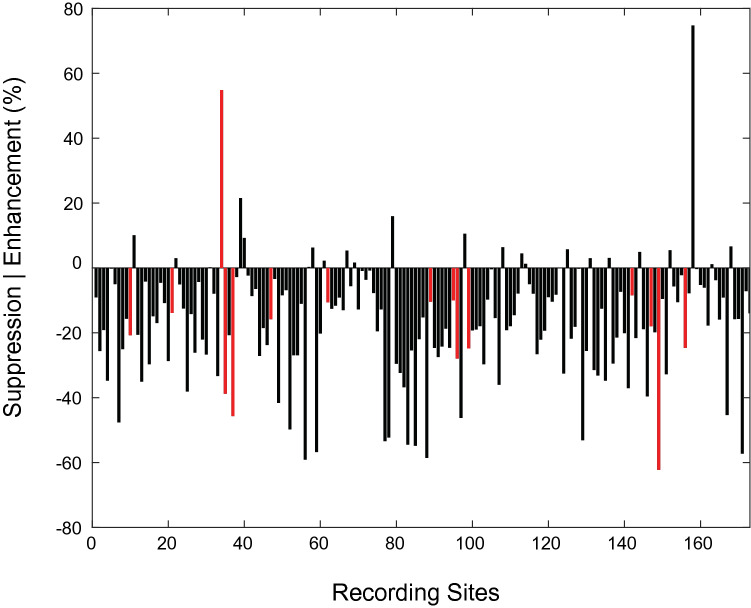
Multisensory interaction indices (INDX1) for evoked responses during the holding period. Red bars indicate recording sites that exhibited statistically significant effects of sensory condition (ANOVA, *p* < 0.05).

Although only a relatively small percentage of individual recording sites (~ 9%, N = 15) exhibited statistically significant effects of sensory condition during the holding period, an analysis of multisensory enhancement/suppression indexes (INDX1; Eq. 1) calculated at each recording site revealed that activity during Bimodal trials was generally suppressed relative to activity on Unimodal trials. Figure 2 shows a bar graph of these indices for all recording sites. For the preferred hand location, over 87% had a negative index value (mean = − 20.6, SD = 18), indicating activity was largely suppressed during Bimodal trials relative to Unimodal trials. Importantly, INDX1 reflects differences in activity between conditions at the preferred location defined by the *Unimodal* condition. Thus, this index assumes that tuning was identical in the two conditions. When tuning was compared between conditions at individual sites, differences were common: 123 recording sites (72%) had different preferred locations for the two sensory conditions (mean difference of ~ 2 locations). Moreover, when sites were grouped by differences in preferred location between sensory conditions, values of INDX1 were observed to be smallest for differences of zero, but were larger for greater differences. More specifically, average indexes (± SD) were − 4.04 (18.99), − 13.18 (17.43), − 25.18 (15.20), − 22.27 (16.60), and − 20.73 (15.62) for differences of 0–4, respectively. These observations support the idea that values of INDX1 partly reflect differences in tuning between conditions. See Fig. 2 in Supplementary materials for more detail.

To account for the effects of tuning changes on multisensory enhancement/suppression, we also calculated an index based on the preferred location in *each* sensory condition (INDX2). Here, suppression was not as common nor as strong (mean index value = − 13.8, SD = 20.3), though still occurred at a majority of recording sites during the Bimodal condition (see Supplementary Fig. 1). In addition, when sites were again grouped by difference in preferred location, INDX2 showed little dependence on spatial differences, with mean values (± SD) of − 3.51 (18.89), 3.98 (22.87), − 2.39 (20.45), − 1.95 (18.94), and 0.17 (21.16) for differences of 0–4. Overall, these findings suggest that differences in LFP activity at individual sites could reflect some combination of spatial and sensory encoding.

**Figure 3:**
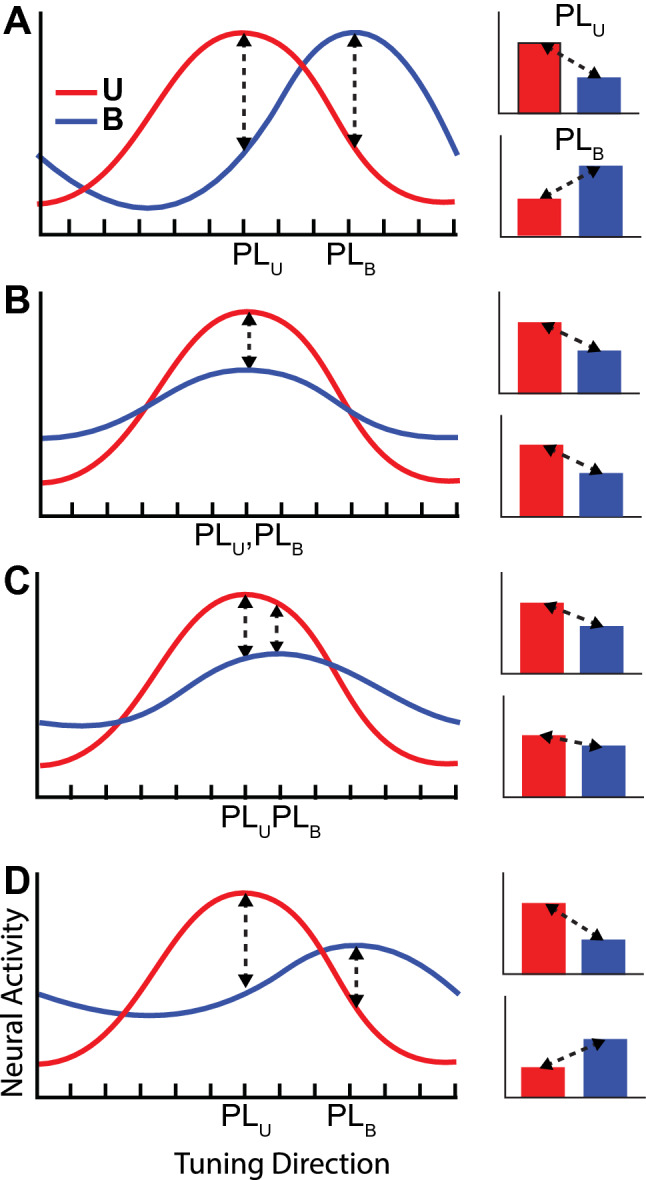
Idealized cosine-tuning curves for hand location in the presence and absence of multisensory suppression and/or tuning changes. Cosine tuning functions were assumed based on (Georgopoulos et al.^39^) (see also (Lalazar et al.^37^). (**A**) Shift in tuning with no suppression. (**B**) Suppression without tuning changes. (**C**) Suppression combined with small tuning changes. (**D**) Suppression combined with large tuning changes. PL_U_ and PL_B_ are preferred locations in the unimodal and bimodal conditions, respectively. See text for details.

Tuning differences, multisensory suppression or a combination of the two effects lead to different predictions regarding population level analyses focused on the preferred direction. For example, Fig. 3 shows idealized tuning curves for hand location for both conditions^37–39^. In Fig. 3A, the curves exhibit tuning differences between conditions without attenuation of responses in the Bimodal condition, i.e. without multisensory suppression. The bar plots to the right show the expected differences in activity when the preferred location for the Unimodal condition (PL_U_) is used to compare responses as well as when the preferred location for the Bimodal condition (PL_B_) is used. Under this scenario, differences in activity at the preferred location are expected to be equivalent in magnitude but opposite in sign for the two comparisons. For Fig. 3B, tuning is assumed to be identical for the two conditions but with suppression of response magnitude in the Bimodal condition. Here, differences in response magnitudes are expected to be equivalent in magnitude *and* sign for comparisons based on both PL_U_ and PL_B_. In Fig. 3C,D, combinations of tuning differences and bimodal suppression are shown. In these scenarios, differences in response magnitudes for the two comparisons are nonequivalent in magnitude with signs that depend on the difference in tuning. For small differences in tuning, signs are expected to be the same (Fig. 3C), while for larger differences they are expected to differ (Fig. 3D). In conclusion, condition-dependent differences in activity can be distinguished from differences in tuning by comparing the magnitude and sign of response differences between PL_U_ sorted and PL_B_ sorted datasets, a strategy that was employed in the analysis of our population evoked potentials and spectra.

**Figure 4:**
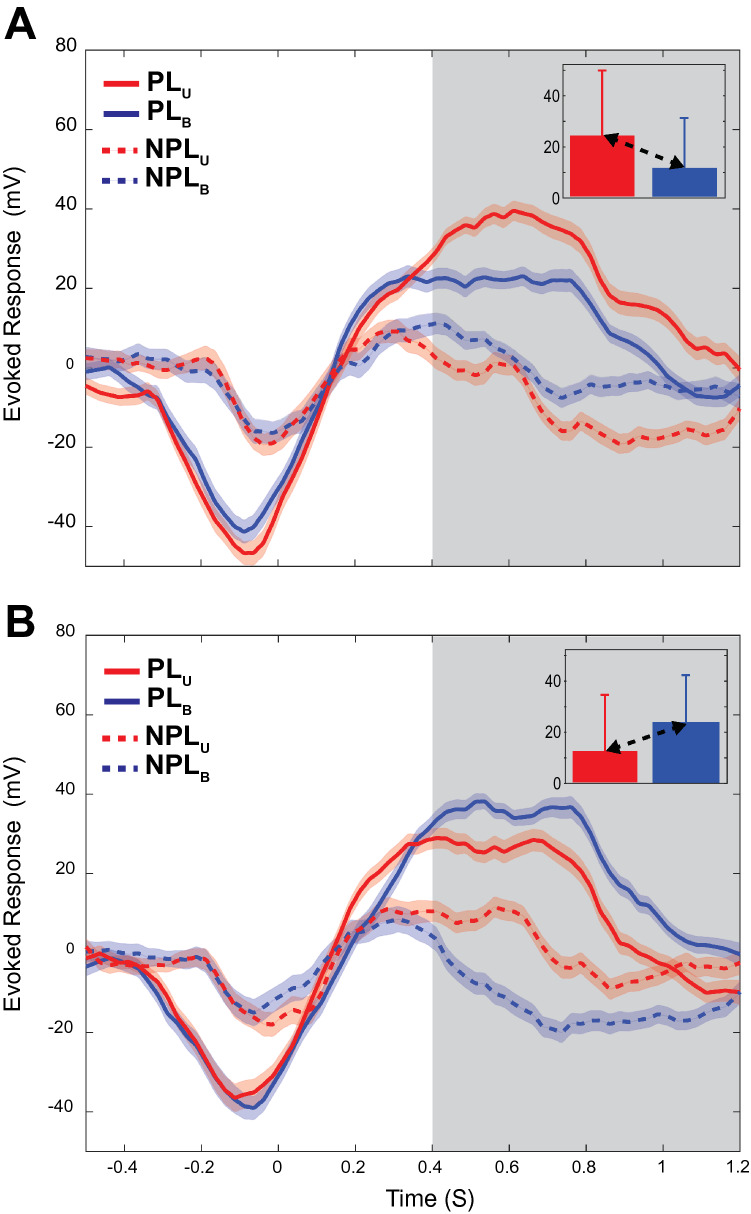
Mean population evoked responses for both sensory conditions and hand locations (preferred and non-preferred). Data are aligned to target acquire (t = 0 s). The grey box (0.4–1.2 s) corresponds to the 800-ms static holding period. U_PL/NPL_ and B_PL/NPL_ refer to unimodal and bimodal trials in the preferred/nonpreferred directions. (**A**) Responses when the Unimodal condition was used to define preferred location (PL_U_). Inset: means and standard deviations over the entire holding period. (**B**) Responses when the Bimodal condition was used (PL_B_).

Figure 4 shows the population evoked responses for PL_U_ sorted (A) and PL_B_ (B) sorted datasets. Activity for both sensory conditions and hand locations (preferred and nonpreferred) are shown. LFP activity prior to movement onset (~ − 0.4 s) was indistinguishable between sensory conditions and hand locations. During the subsequent movement period, the temporal profiles for each sensory condition were largely similar for their respective reach directions, though response magnitudes differed between directions (as expected). Although responses were sorted into preferred or nonpreferred based on activity during the *holding* period, these earlier differences in magnitude indicate that the evoked LFPs were also strongly modulated by movement direction (see also Supplementary Table 1). Notably though, no differences between sensory conditions were observed prior to or during movement.

Near the start of the static holding period (Fig. 4A, grey boxes) differences in evoked responses due to hand location *and* sensory condition became apparent. The divergence of activity between the Unimodal and Bimodal conditions at the start of the holding period is notable because it is the only part of the task where the sensory feedback conditions differed. Previous analyses established that the distributions of hand locations were largely similar between conditions during this period of the task^14^, thus the differences in LFP activity seen here could be attributed to differences in sensory condition, differences in tuning, or both (as previously discussed). For the PL_U_ sorted data (Fig. 4A), evoked activity for the preferred hand location differed significantly between Unimodal and Bimodal conditions during the holding period (*t* test, *p* = 2.05e−11, N = 170), with suppressed activity for the Bimodal condition. For the nonpreferred hand location, activity also differed between conditions during the holding period (*t* test, *p* = 3.23e−09, N = 170) with activity during the Bimodal condition appearing greater (less negative) than that in the Unimodal condition.

For the PL_B_ sorted data (Fig. 4B), the opposite trends were observed. Although activity also differed significantly between the U and Bimodal conditions for both the preferred and non-preferred locations (*t* tests, *p* = 2.82e−11, N = 170), activity in the Bimodal condition appeared *greater* than activity in the Unimodal condition for the preferred location and was more negative in the nonpreferred location. When the unsigned differences in activity between conditions were compared statistically between PL_U_ sorted and PL_B_ sorted datasets, no significant differences were found (*t* tests, *p* = 0.39, N = 170). Thus, at the population level, differences in evoked activity between conditions reflected mainly differences in tuning and not multisensory suppression (scenario in Fig. 3A).

### Frequency domain

**Figure 5:**
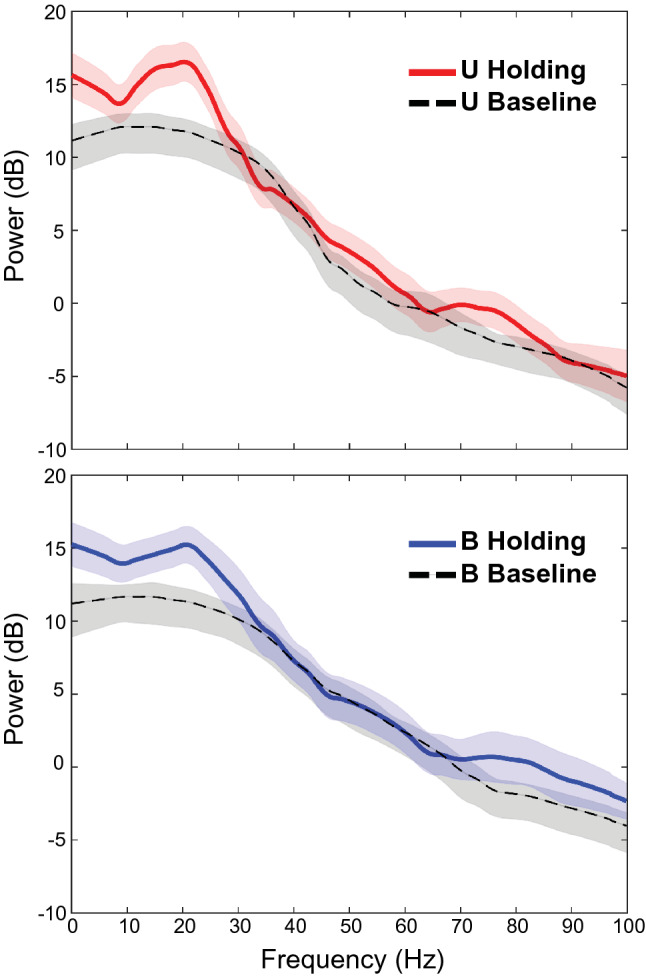
Trial-averaged LFP power spectra (N = 5, with jackknife error bars) for the holding and baseline periods at a single site. Power was enhanced relative to baseline during the holding period from frequencies less than ~ 30 Hz.

Previous results from this area showed that during the maintenance of static arm positions, the spike trains of many neurons were strongly oscillatory in the beta band (13–30 Hz), though modulation of spike timing within other frequency bands was observed as well^17^. As a result, in the present study we analyzed the delta, theta, alpha, beta, and gamma bands of the LFP during the holding period. Figure 5 shows example single site LFP power spectra for the preferred hand location. Spectra obtained during the baseline period (black) are superimposed on spectra obtained during the holding period for both sensory conditions. The shapes of the holding period spectra seen in this figure were typical, with the greatest power being concentrated at the lower frequencies and with a notable ‘bump’ in the beta band. Importantly, power in the lower frequency bands (delta, theta, alpha, beta) generally increased during the holding period relative to that in the baseline period, regardless of sensory condition or final hand location. Nevertheless, power in these frequency bands was modulated by sensory condition and/or hand location at some sites, as described below.

A two-way ANOVA was used to quantify the effects of sensory condition and final hand location on LFP power. Responses across recording sites were largely similar between sensory conditions and hand locations, with many sites having no main effects of either factor, especially in the delta, theta, and alpha bands (see Table 3 in Supplementary Materials). Hand location generally had a greater influence on spectral power than did sensory condition during the holding period, particularly at higher frequencies (beta and gamma), where approximately 20% of the sites showed statistically significant effects.

**Figure 6:**
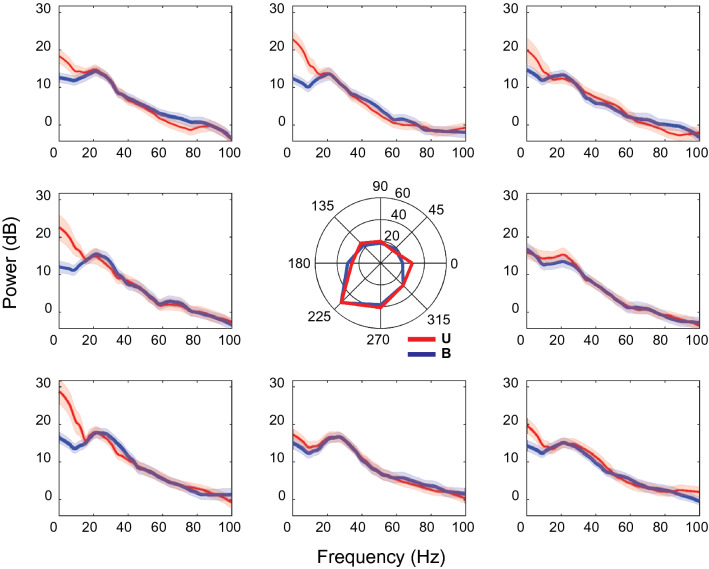
Trial-averaged LFP spectra (with jackknife error bars) from a single recording site that showed main effects of hand location in the beta and gamma bands during the holding period. Spectra were averaged across trials (N = 5). Blue traces are Bimodal trials, red traces are Unimodal trials. Spectral power is in log units. Central polar plot is based on mean power in beta band.

Figure 6 shows the power spectra for an example recording site that showed main effects of hand location in the beta and gamma bands. At this site, beta power was tuned for hand locations down and to the left of the starting position, i.e. toward targets at 225°. However, no differences in power between sensory conditions were observed for this frequency band. This was confirmed by ANOVA, which showed a statistically significant effect of hand location *(F* = 9.46, *p* = 5.3e−8), but no main effect of sensory condition (*F* = 0.21, *p* = 0.65) nor interaction effects (*F* = 0.29, *p* = 0.96). Interestingly, at this site power in the lower frequencies was not tuned to direction but tended instead to show effects of the sensory conditions. Power in the alpha band was not tuned to final hand location (*F* = 1.02, *p* = 0.43) but did differ significantly between sensory conditions (*F* = 5.85, *p* = 0.019), being stronger for the Unimodal condition. No significant interaction effect was found (*F* = 0.59, *p* = 0.77). Thus, when effects of the sensory conditions were observed at single sites they were not necessarily coupled to effects of hand location.

**Figure 7:**
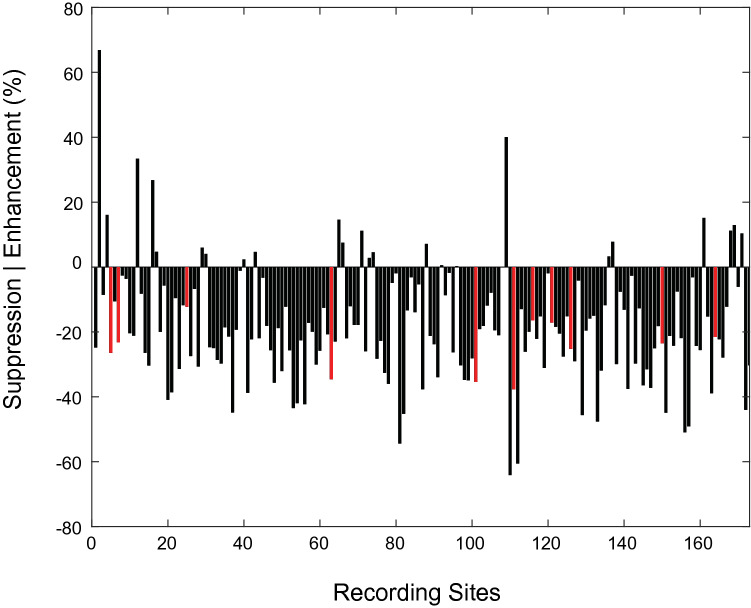
Multisensory interaction indices (Eq. 1) for beta power during the holding period. Red bars indicate recording sites that exhibited statistically significant effects of sensory condition (ANOVA, *p* < 0.05).

As with the evoked activity, even though only a relatively small percentage of individual recording sites (6%, N = 10) exhibited statistically significant effects of sensory condition on beta power during the holding period, power was generally suppressed on Bimodal trials relative to power on Unimodal trials (INDX1, Fig. 7). Over 86% of the sites had a negative index value (mean = − 20.45, SD = 13.49), indicating that beta power was generally greater on Unimodal trials than Bimodal trials. As with the evoked responses, when sites were grouped by difference in preferred location, INDX1 values were observed to be smallest for differences of zero, and were larger for greater differences (mean (± SD): − 6.1 (12.39), − 24.76 (16.63), − 22.99 (13.95), − 23.75 (12.63), and − 21.03 (11.62) for differences of 0–4, respectively). These observations support the idea that for beta band activity, values of INDX1 partly reflect differences in tuning between conditions. See Fig. 3 in Supplementary materials for more detail.

To further assess the extent to which tuning differences between conditions might have played a role in this apparent suppression, we also calculated multisensory enhancement/suppression indices using Eq. 2 (INDX2), and found suppression was not as common nor as strong (median index value = − 3.45). In addition, INDX2 showed little dependence on differences in preferred location, with mean (± SD) values of − 2.97 (14.08), − 4.33 (12.7), − 2.39 (13.8), − 2.3 (13.23), and − 3.64 (12.16) for differences of 0–4. Overall, these findings suggest that differences in beta power at many sites reflected either tuning differences alone or in combination with multisensory suppression.

**Figure 8:**
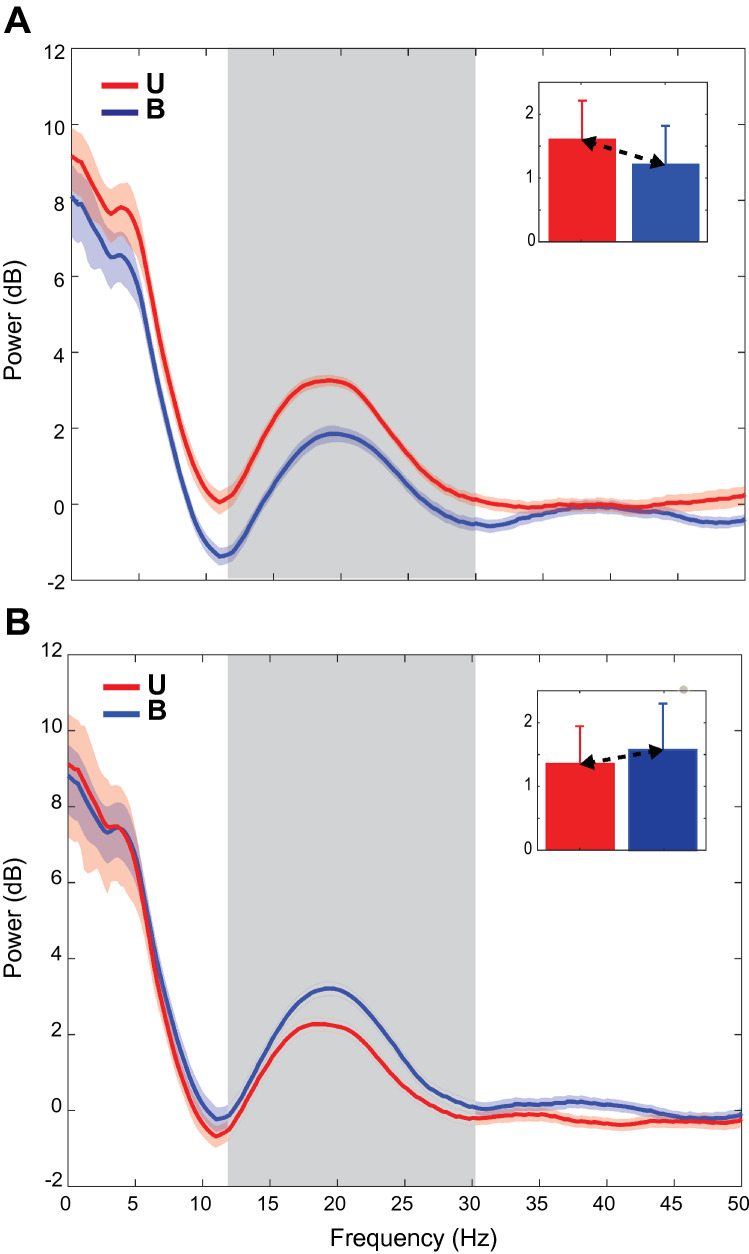
Population spectra for all recording sites in the B (blue) and U (red) conditions during the holding period for the preferred direction. Error bars are jackknife error bars. The grey box indicates the beta band (13–30 Hz). U_PL/NPL_ and B_PL/NPL_ refer to unimodal and bimodal trials in the preferred/nonpreferred directions. (**A**) Spectra when the Unimodal condition was used to define preferred location (PL_U_). Inset: means and standard deviations of the power over the beta band. (**B**) Spectra when the Bimodal condition was used (PL_B_).

Figure 8 shows the population averaged spectra during the holding period for PL_U_ sorted (Fig. 8A) and PL_B_ sorted (Fig. 8B) datasets. Activity for both sensory conditions and the preferred hand location (based on beta power) are shown. Power during the holding period was largely concentrated in the lower frequency bands, with the greatest power being observed in the delta range of frequencies. Power in the theta and alpha frequencies dropped sharply before rising again during the beta band—consistent with the beta bump seen in the single site spectra (Figs. 5, 6).

For the PL_U_ sorted data (Fig. 8A), power in the beta band differed significantly between Unimodal and Bimodal conditions during the holding period (*t* test, *p* = 1.55e−15), with activity for the Bimodal condition appearing suppressed with respect to the Unimodal condition. Power also differed significantly between the Unimodal and Bimodal conditions for the PL_B_ sorted data (Fig. 8B; *t* test, *p* = 2.35e−5), but in this case power in the Bimodal condition was greater than activity in the Unimodal condition. Critically, and in contrast to the evoked potentials, unsigned differences in beta power between conditions *differed* between the PL_U_ and PL_B_ sorted datasets (*t* tests, *p* = 0.03). Thus, at the population level, beta power was largely consistent with scenario *D* in Fig. 3, indicating that differences in power between conditions reflected both differences in tuning *and* an overall attenuation of responses in the Bimodal condition (multisensory suppression).

## Discussion

Here we examined the effects of bimodal (visual-proprioceptive) interactions on LFP signals recorded at multiple sites in the SPL as non-human primates performed an arm position maintenance task. We found that the effects of multisensory interactions on LFP activity (evoked and beta band) were dependent in part upon the criteria used to define preferred hand location. When activity on Unimodal trials was used to define the preferred location, multisensory interactions appeared to result predominantly in suppression. However, when activity for both types of trials was used, suppressive effects weakened or reversed sign at many individual sites. At the population level, differences in evoked activity between conditions appeared to result largely from feedback-dependent tuning to hand location. However, for beta band power, effects were more consistent with multisensory suppression superimposed upon feedback-dependent tuning differences. This suggests that different aspects of arm position maintenance (spatial location vs sensory feedback signals available for position) are coupled within the frequency domain representations of LFP activity in the SPL and can potentially be decoded to extract the current location of the limb, regardless of sensory conditions. More generally, the results of this study indicate that neural activity related to arm position in the SPL is differentially modulated by available sensory feedback, with these feedback-dependent differences being observable in both the time and frequency domains. Precisely how different combinations of feedback are translated into a unified representation of arm position remains unclear however, and will require further investigation.

In the time-domain, evoked activity was suppressed on Bimodal trials in a manner similar to spiking activity^14^, both in degree and in the extent to which tuning differences contributed to this apparent suppression. Previous work has shown that LFP activity and spiking activity are highly correlated in several cortical areas^40–43^, though the precise nature of the relationship between these signals remains controversial^44–46^. For example, although many studies have assumed that LFPs and spikes represent separate components of extracellular signals, a recent study of the medial temporal area MT showed that LFP activity peaks later than, is partially predicted by, preceding spiking activity, suggesting that modulations of lower frequency LFPs are an epiphenomena of local spiking^47^. However, the extent to which local spiking contributes to LFPs may be brain area- and brain state–dependent^44^, thus the precise nature of the relationship between spikes and LFPs in the PPC during arm position maintenance remains equivocal and a potential focus of future studies.

For the frequency-domain, we expected to see enhanced beta band spectral power at individual sites for only one or both sets of feedback conditions, as was previously observed for spiking activity^17^. Instead we found that enhanced beta power was ubiquitous across recording sites regardless of sensory condition and final hand location. Moreover, few individual sites showed effects of sensory condition on beta power, though at the population level multisensory suppression of this enhanced beta band activity was observed. What mechanism could account for the prevalence of feedback-independent enhancement of LFP beta power, but *site and feedback-dependent* enhancement of beta power in spiking activity? One possible explanation relates to differences in connectivity patterns of neurons within a given cortical area. Cortical areas receiving strong sensory inputs are thought to contain multiple interconnected subnetworks of neurons that may be selectively responsive to sensory features^48^. However, all ionic processes (such as transmembrane synaptic inputs) contribute to the extracellular electrical field^49,50^. Thus in a given cortical area, the spiking activity of individual neurons may be dependent on their subnetwork-specific responses to selective task conditions, whereas the LFP signal reflects the activity of any of the overlapping subnetworks engaged by a task. Furthermore, spiking activity is dependent on the unique biophysical properties of the neurons within a particular subnetwork, and this may influence which neurons are ultimately entrained by oscillatory synaptic input^51^.

One of the overriding principles of MSI, based on numerous behavioral and computation studies, is that that sensory cues are weighed according to their relative reliabilities in a given context^52^. The experiments described here did not systematically alter the reliability of either visual or proprioceptive inputs, thus it is difficult to determine the extent to which the suppression of beta band activity at the population observed here can be attributed to bottom-up, stimulus-driven processes. However, MSI is influenced not only by relative signal reliabilities but also top-down attentional processes^53–55^. Effects of bottom up versus top down processes on MSI have been observed to be frequency dependent, with bottom-up processing being reflected in frequency bands greater than 30 Hz (e.g. gamma band), and top-down processing reflected in lower bands such as beta^53,56^. Specific effects such as enhancement or suppression may also be task and area/network dependent. For example, in an audio-visual congruence task, found that attention led to *increased* gamma band activity but *decreased* beta-band activity in early sensory cortex areas^57^.

Effects of endogenous attention on MSI have particular relevance to the current results. The data reported here were obtained from recordings of the superficial cortex of the SPL, an area that is believed to be strong proprioceptive inputs from primary somatosensory areas^58–60^. On Unimoda trials, beta band LFP was elevated relative to that on Bimodal trials. This could reflect the fact that in order to maintain position under these conditions, animals needed to strongly attend to signals provided by the proprioceptive (and motor) systems. On Bimodal trials, where vision was also available for position monitoring, attention to proprioception was likely not as critical. Thus, the reduction in beta activity could reflect an attention-driven shift in the balance of activation among cortical areas involved in position monitoring, from those that are more dominantly proprioceptive to those that are more visual in nature^54^.

Evoked activity and beta band power were tuned to arm location at many individual sites and at the population level, consistent with evidence that the SPL in primates is involved in the multisensory representation of arm position^14,17,24,61,62^ and arm postural control^61,63–65^. Critically, however, tuning to hand location often differed between unimodal and bimodal trials. Tuning is not currently believed to be a static feature of neural responses, as tuning curves of individual neurons can change as a function of time, learning, attention, and differences in kinematics/dynamics, among other factors^66–73^. In the current experiments, animal’s performed a task on which they were highly trained, thus it is unlikely that learning related factors contributed strongly to condition-dependent tuning differences. In addition, data were analyzed during the same ‘holding’ epoch for both conditions, and neither mean arm position nor position variability differed between conditions. Although it is conceivable that, due to the kinematic redundancy of the arm, different arm configurations were used for the same location in the two conditions, these differences were likely slight, thus it is also unlikely that biomechanical factors contributed strongly to tuning differences between conditions. Lastly, it’s unlikely that the tuning changes observed here were due to temporal factors, as the conditions were run concurrently and were randomly interleaved on a trial by trial basis. This suggests that multisensory interactions altered the tuning properties of some sites (and neurons^14^) in the SPL on very short time scales in these experiments.

What could account then for the observed differences in tuning between sensory conditions? As discussed above, attention-driven shifts in the balance of activation among cortical areas could be responsible for the multisensory suppression of beta band activity observed here, thus the notion that tuning differences were also drive by attention is certainly plausible. Another possibility is that differences in tuning could reflect context-dependent encoding of hand position^74,75^. That is, in the absence of continuous and reliable visual feedback (e.g., for movements generated in the dark or following long delays without feedback) hand location is determined largely by proprioceptive feedback (and/or forward modeling) and is therefore likely to be encoded in a body-centered frame of reference. In contrast, when the hand is visible, hand location could be remapped from body to eye-centered coordinates, or be encoded in both reference frames simultaneously. Evidence from previous studies focusing on the SPL and dorsal premotor cortex are consistent with the idea that arm movement and posture related variables can be encoded in multiple frames of reference simultaneously^76–78^, thus it is conceivable that a similar phenomenon underlies the feedback-specific tuning observed here.

## Supplementary information

Supplementary information
