# Supplementary material for: Sensory feedback-dependent coding of arm position in local field potentials of the posterior parietal cortex
Source: Sci Rep. 2021 Apr 27;11:9060. doi: 10.1038/s41598-021-88278-5 (PMC8079385; doi:10.1038/s41598-021-88278-5)
Supplement: Supplementary file 1 — Supplementary information [file 41598_2021_88278_MOESM1_ESM.docx]

*Title:* Sensory feedback-dependent coding of arm position in local field potentials of the posterior parietal cortex

*Authors:* Paul VanGilder^1^, Ying Shi^1^, Gregory Apker^1^, Christopher A. Buneo^1^*

*^1^*School of Biological and Health Systems Engineering, Arizona State University, Tempe, AZ

**Corresponding Author:* C. A. Buneo, School of Biological and Health Systems Engineering, Arizona State University, P.O. Box 879709, Tempe, AZ 85287-9709, U.S.A. E-mail: cbuneo@asu.edu

We used a two-way ANOVA to examine the effects of hand location and/or sensory condition on the evoked LFP response across recording sites. The results are summarized in Supplementary Table 1, which indicates the percentage of recording sites (N=170) that exhibited statistically significant effects. In general, evoked LFPs were modulated more by hand location than visual condition. Significant effects of hand location during the movement and holding periods were observed at 57 (33%) and 43 (25%) of recording sites, respectively. Effects of sensory condition (unimodal vs. bimodal condition) and interaction effects were much less common.

| Results of two-way ANOVA on Evoked Responses | | | |
| --- | --- | --- | --- |
|  | Baseline | Movement | Holding |
| Hand Location | 3% | 33% | 25% |
| Sensory Condition | 6% | 5% | 9% |
| Interaction | 2% | 2% | 3% |

Supplementary Table 1: Summary of two-way ANOVA on baseline, movement, and hold epochs. Numbers represent percentages of recording sites (n=173) that showed significant effects (p<0.05).

In addition to performing a two-way ANOVA (factors: hand location, sensory condition), we also examined the effects of hand location on evoked responses for each sensory condition individually using one-way ANOVAs. Those analyses were performed for both the movement and holding epochs and are summarized in Supplementary Table 2.

| Significant Effects of Hand Location | | |
| --- | --- | --- |
|  | Movement | Holding |
| Unimodal | 23% | 16% |
| Bimodal | 29% | 16% |
| Both | 15% | 6% |

Supplementary Table 2: Summary of ANOVA results for significant effects of hand location for the movement and holding epochs in each sensory condition.

For the evoked responses, we computed the enhancement/suppression interaction index using a second equation that took into account the preferred location in the bimodal condition. The results of that alternative analysis are presented here in Supplementary Figure 1.

Supplementary Figure 1 Enahancement and suppression index values of the evoked LFP response calculated using Equation 2.

Suppression | Enhancement

For the frequency domain data, a two-way ANOVA was used to examine effects of hand location and/or visual condition on mean power across the delta, theta, alpha, beta, and gamma bands. Supplementary Table 2 summarizes these results. A majority of sites showed no significant effects, but in general, a greater number of sites exhibited significant differences in mean power in response to hand location rather than sensory condition. This was particularly true for the beta and gamma bands, in which 36 and 42 sites, respectively, showed main effects of hand location.

| Results of two-way ANOVA on LFP Spectra | | | | | |
| --- | --- | --- | --- | --- | --- |
|  | Delta | Theta | Alpha | Beta | Gamma |
| Hand Location | 8% | 6% | 12% | 21% | 24% |
| Sensory Condition | 6% | 6% | 6% | 6% | 5% |
| Interaction | 3% | 2% | 2% | 5% | 3% |

Supplementary Table 3: Summary of two-way ANOVA on baseline, movement, and hold epochs. Numbers represent percentages of recording sites (n=173) that showed significant effects (p<0.05).

To test if spectral power was modulated by hand location during unimodal, bimodal, or both sensory conditions, we performed one-way ANOVAs on mean spectral power for each frequency band during the static holding period. In general, fewer recording sets showed effects of hand location in the one-way ANOVA compared to two-way ANOVA (factors: hand location, sensory condition). However, the one-way ANOVA does not take into account any interaction effects, so for our main analysis we included only the two-way ANOVA results.

Supplementary Table 4: 1 One-way ANOVA results for effects of hand location in either or both sensory conditions for mean power during the holding period for each frequency band.

| Significant Effects of Hand Location | | | | | |
| --- | --- | --- | --- | --- | --- |
|  | Delta | Theta | Alpha | Beta | Gamma |
| Unimodal | 7% | 5% | 7% | 12% | 21% |
| Bimodal | 8% | 4% | 6% | 14% | 13% |
| Both | 1% | 1% | 1% | 3% | 10% |

We also addressed the change in spatial tuning (i.e. preferred locations) between the sensory conditions. The spatial locations of the reach targets were identical for both conditions, and a previous analysis of the behavior showed that mean reach endpoint was nearly identical between the two conditions. The mean percentage of recording sessions across both animals (N=343) and targets (N=8) with a significant difference in reach endpoint was 6.9±1.5% (MANOVA, P<0.05), thus we have interpreted the changes in neural activity to be due to differences in sensory condition and/or spatial tuning, and not differences in overt behavior.

To determine whether the degree of suppression (quantified by INDX1 and INDX2) was correlated in some way with the degree of spatial tuning difference between conditions, we looked at whether the index values decreased (i.e. greater suppression) as the difference between preferred locations got larger. To review, on each trial, the animals made center-out reaches from a starting position to one of eight peripheral targets. The preferred location for the unimodal (PL_U_) and bimodal (PL_B_) conditions were defined as the target with the largest trial-averaged evoked or spectral response for each recording site. We defined the tuning difference as the difference in preferred location between sensory conditions, i.e. the number of target locations between PL_U_ and PL_B_, as measured by the shortest distance clockwise or counter-clockwise around the 8 target locations. Thus, the maximum difference between preferred locations could never exceed four. We then sorted the enhancement/suppression indices based on the difference in spatial tuning. Supplementary Figure 2 shows the suppression values (INDX1) for all possible differences in preferred location between sensory conditions. Differences of zero (no difference in spatial tuning between conditions) showed the smallest degree of suppression. Larger differences in preferred location were associated with progressively larger degrees of suppression (though this effect plateaued a difference of 2 locations). Average indexes were -4.04, -13.18, -25.18, -22.27, and -20.73 for differences of 0-4, respectively. These observations support the idea that values of INDX1 partly reflect differences in tuning between conditions.


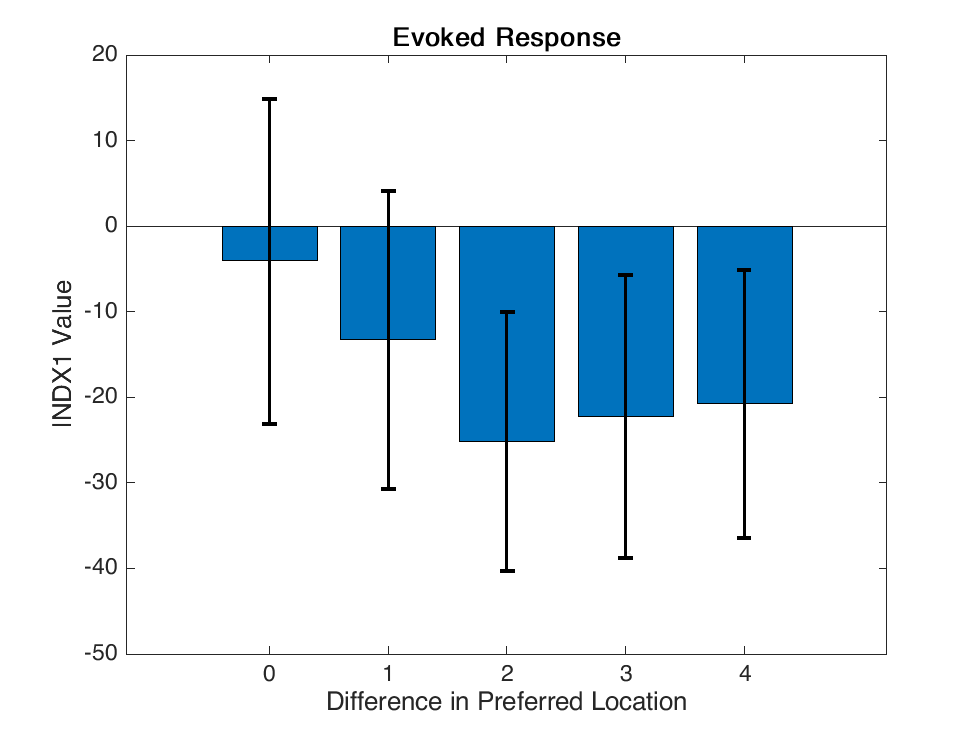


Supplementary Figure 2: Comparison of the degree of suppression of the evoked response as a function of spatial tuning for all recording sites. Bar graph is mean index value (INDX1) quantifying multisensory enhancement/suppression sorted by difference in preferred location between sensory conditions. Negative values indicate response suppression. Differences in spatial tuning along the x-axis are the number of reach targets separating PL_U_ and PL_B_. Error bars indicate population standard deviation.

We performed a similar analysis for the mean Beta power As with the evoked responses, when sites were grouped by difference in preferred location, INDX1 values were observed to be smallest for differences of zero, and were larger for greater differences (mean: -6.1, -24.76, -22.99, -23.75, and -21.03 for differences of 0-4, respectively). These observations support the idea that for beta band activity, values of INDX1 partly reflect differences in tuning between conditions.


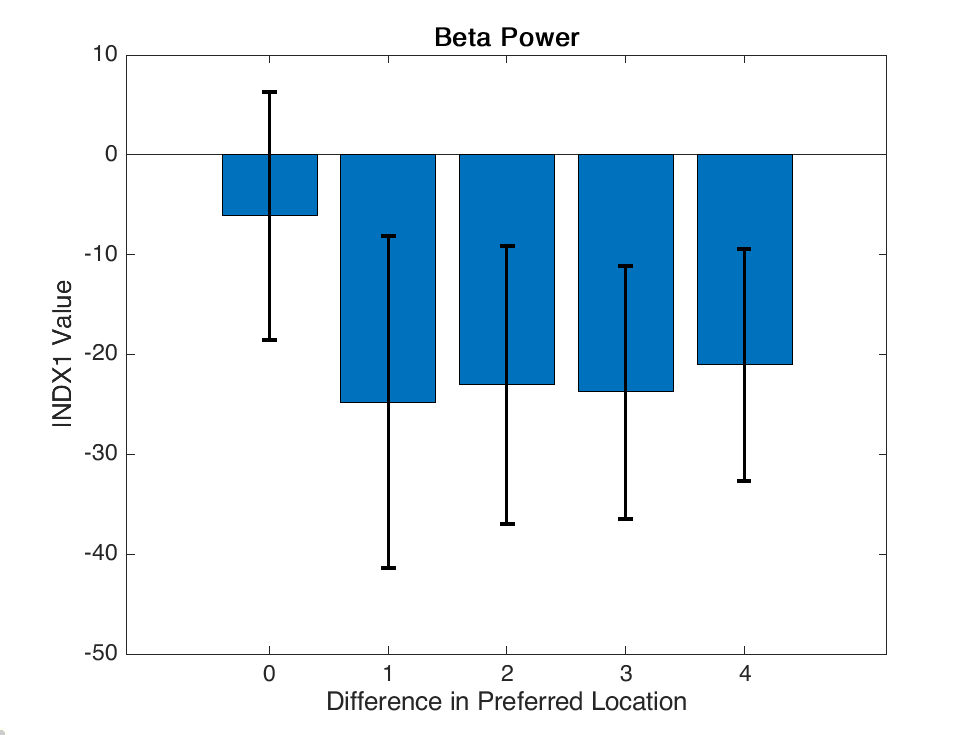


Supplementary Figure 3: Comparison of the degree of suppression of the mean Beta power as a function of spatial tuning for all recording sites. Bar graph is mean index value (INDX1) quantifying multisensory enhancement/suppression sorted by difference in preferred location between sensory conditions. Negative values indicate response suppression. Differences in spatial tuning along the x-axis are the number of reach targets separating PL_U_ and PL_B_. Error bars indicate population standard deviation.
